# Harnessing the Topography of 3D Spongy-Like Electrospun Bundled Fibrous Scaffold via a Sharply Inclined Array Collector

**DOI:** 10.3390/polym11091444

**Published:** 2019-09-03

**Authors:** Sun Hee Cho, Jeong In Kim, Cheol Sang Kim, Chan Hee Park, In Gi Kim

**Affiliations:** 1 Department of Bionanosystem Engineering, Graduate School, Chonbuk National University, Jeonju 54896, Korea; 2 Division of Mechanical Design Engineering, College of Engineering, Chonbuk National University, Jeonju 54896, Korea

**Keywords:** electrospinning, fiber topography, cottony fibers, 3D electrospun scaffolds, 3D aligned fibers

## Abstract

To date, many researchers have studied a considerable number of three-dimensional (3D) cotton-like electrospun scaffolds for tissue engineering, including the generation of bone, cartilage, and skin tissue. Although numerous 3D electrospun fibrous matrixes have been successfully developed, additional research is needed to produce 3D patterned and sophisticated structures. The development of 3D fibrous matrixes with patterned and sophisticated structures (FM-PSS) capable of mimicking the extracellular matrix (ECM) is important for advancing tissue engineering. Because modulating nano to microscale features of the 3D fibrous scaffold to control the ambient microenvironment of target tissue cells can play a pivotal role in inducing tissue morphogenesis after transplantation in a living system. To achieve this objective, the 3D FM-PSSs were successfully generated by the electrospinning using a directional change of the sharply inclined array collector. The 3D FM-PSSs overcome the current limitations of conventional electrospun cotton-type 3D matrixes of random fibers.

## 1. Introduction

Cells can be inherently sensitive to ambient microenvironment such as nano to microscale fibrous patterns of topography [1]. For this reason, there are many tissue engineering approaches attempting to address this issue through the presentation of topographical cues which precisely mimic the native ECM to regulate the cell function and control the resulting tissues construct [2,3]. Fibrous matrixes manufactured by electrospinning technique have emerged as one of the powerful tools to present the topographic cues because of their structural similarity to the native ECM and collagen fibers, thus making them a suitable material to guide the formation of new tissue. Although the fiber whipping instability in the electrospinning process makes it difficult to control the deposition location of the fiber and the resultant sophisticated and patterned structure, a lot of efforts have been conducted to the capability in the development of patterned fibers in the forms of precise positioning. But the efforts still suffer from technical challenges associated with the fabrication of the 3D fibrous electrospun matrixes with well-defined structures to recapitulate the natural ECM characteristics. Cai et al. demonstrated electrospun matrixes with fibers oriented randomly and evenly in 3D were formed by the electrostatic repulsion between fibers [4]. Their results of in-vitro study have shown that cells cultured on the developed 3D electrospun matrix develop into stereomorphic topography instead of being flattened cells when cultured on conventional two-dimensional (2D) electrospun membranes [3,5]. Although recent studies have attempted to generate 3D electrospun matrixes based on the fiber repulsion, despite the fluffy and low-density fibrous appearance of the matrixes, the resulting 3D matrixes fabricated by repulsion force still contains planar membranes and failed to control the topography of the fibers in the matrix.

In this study, scaffolds with fibers evenly patterned in 3D were successfully fabricated with the aid of lactic acid (LA) based on the principle of electrostatic repulsion. Furthermore, unique constructs of the scaffolds are different from conventional 3D electrospun scaffolds with random fibers. The developed 3D FM-PSS attempts to mimic the native ECM, and the mechanism has been proposed and validated to enable control of the fiber topography of the scaffold. 

## 2. Experimental Procedure

**Figure 1:**
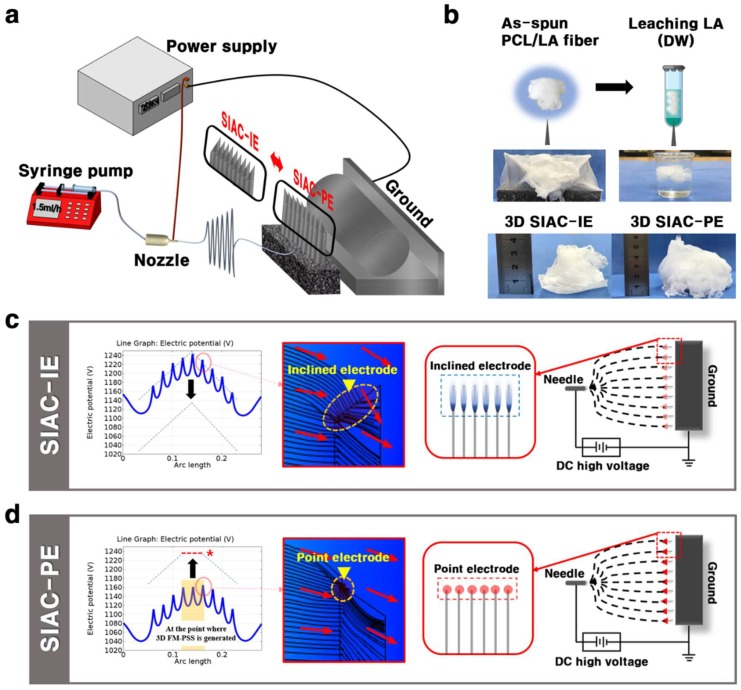
(**a**) Schematic illustration of electrospinning set up. (**b**) LA leaching process of PCL/LA fibers. Electric field analysis results of (**c**) SIAC-IE and (**d**) SIAC-PE.

Polycaprolactone (PCL, Sigma-Aldrich Korea, Korea) and LA solution (Showa, Japan) were used for the fabrication of the 3D FM-PSS. Dichloromethane (DCM) and N,N-dimethylformamide (DMF) were purchased from Samchun, Korea. PCL solution was prepared by dissolving PCL pellets (10 wt %) in a blended solvent of DCM/DMF (4:1, *w*/*w*). LA was added in 10 wt % PCL solution at loads corresponding to 15 wt % (based on total mixture weight). Then, the prepared solution was stirred for 6 h and kept for 24–48 h at room temperature. The 3D FM-PSS was fabricated using the sharply inclined array collector (SIAC) consist of nine edged bars and pedestal as shown in Figure 1a. Electrospinning parameters were set as follows: flow rate of 1.5 mL/h, voltage 20 kV, and needle to SIAC distance of 12 cm. The electric field simulation for the electrospinning was performed using COMSOL^®^Ver.5.0. The morphological analysis of the 3D FM-PSS was conducted by scanning electron microscopy (SEM, Hitachi, Japan) and Image J program (NIH, Bethesda, MD, USA). Viscosity was measured by digital rheometer (Brookfield, DV-3+, Boulevard, Middleboro, MA, USA). The fabricated 3D fibrous scaffolds were then washed in distilled water (DW) for 24 h to remove the residual LA. The samples were prepared before and after the LA leaching for further analysis and comparison of the samples. The presence of LA in the 3D FM-PSS was determined by Fourier transform infrared spectroscopy (FT-IR, Perkin Elmer, Waltham, MA, USA), Thermogravimetric Analyzer (TGA, TA Instruments Ltd., Waltham, MA, USA), and Differential Scanning Calorimeter (DSC, TA Instruments Ltd., New Castle, DE, USA). The porosity of the samples was evaluated using SEM images and Image J software program. The dog-bone shaped FM-PSS sample (according to American Society for Testing and Materials (ASTM) standard D638, the sample was prepared in type V) was prepared, and the tensile test was carried out on a universal testing machine (MTDI INC, Korea) at a 10N KAF-TC load sensor with a strain rate of 5 mm/min at room temperature. For cell viability assay, the 3D FM-PSS was prepared and sterilized under ultraviolet (UV) radiation for 24 h. The MC3T3-E1 cells were cultured on the FM-PSS in 48-well plate at a density of 2 × 10^4^ cells. The alpha-Eagle’s minimal essential medium (α-MEM) supplemented with 1% penicillin-streptomycin and 10% fetal bovine serum (FBS) was used for the cell culture. All the culture regent was obtained from Hyclone (Logan, UT, USA). The morphology and infiltration of cells were examined using confocal laser scanning microscope. 

## 3. Results and Discussion

The details of the electrode configuration can affect the electric fields which leads to changes in the deposition of the fibers, resulting in different topography of fibers. In this study, we designed the novel electrospinning set up to confirm that the fiber morphology of the 3D fluffy fibrous scaffold varies depending on the direction of the edged bars (Supporting experimental section and Movie 1). The electrospinning set up contains a device of SIAC which consists of the nine same edged bars (thickness 0.37 mm, length 65 mm) and pedestal as shown in Figure 1a. The detailed information of the edged bars is shown in Figure S1. The procedure used to fabricate the cotton-type 3D FM-PSS involves the generation of the cotton-type PCL/LA 3D FM-PSS followed by selective removal of the LA (Figure 1b). The result indicates that incorporating LA into PCL assisted the generation of the PCL/LA fluffy constructs due to the electrostatic repulsion by the carboxylic acid functional groups in LA [6,7,8]. The negatively charged carboxyl group of LA in the PCL/LA fibers under the influence of the increasing electric field can result in repulsive forces between fibers, generating 3D cotton type fibrous matrixes. However, the residual LA in the fibers should be removed before implanting. The purpose to leach LA was that it increases the acidity of the scaffold, which can induce cell injury. In addition, the presence of LA accelerates the degradation of PCL fibers. The morphological variation of the as-spun PCL/LA fiber with residual LA is shown in Figure S2. For these reasons, the LA leaching process is required to preserve the physical properties of the as-spun PCL/LA fiber. The fabricated 3D fibrous scaffolds were washed in DW for 24 h to remove the residual LA.

The software of COMSOL^®^ Multiphysics was utilized to guide the actual electrospinning set up for the analysis of the effects of the electric fields on the fiber morphology. The effects of the position of the edged bars on the intensity of the electric potential are presented in Figure 1c. In both method setting, the electric potential direction changes repeatedly in the positive (+) and the negative (−) directions depending on the presence or absence of the edged bars. In the electric potential analysis graph of the setting SIAC with point electrode (SIAC-PE), the peak-to-valley ratio of the portion where the 3D fibers were generated (Figure S3) was uniform in accordance with the conditions required to fabricate aligned fibers (represented by red dotted line with a star in Figure 1c). However, in the electric potential analysis graph of the setting SIAC with inclined electrode (SIAC-IE), the peak and valley values continuously changed (from 1150 V (minimum value) to 1240 V (maximum value)). The continuous fluctuation of the electric fields generated 3D random fibers because of the increased instability of the fibers [9]. By controlling the position of the edged bars, so-called “point electrode” and “inclined electrode” configurations are designed in this study; in the former, the electric field lines are strongly curved at the point electrode, whereas, in the latter configuration, the electric field is comparatively uniform at the line electrode (represented by yellow arrow in Figure 1c,d). This result was verified by electrical streamline simulation as shown in Figure S4.

**Figure 2:**
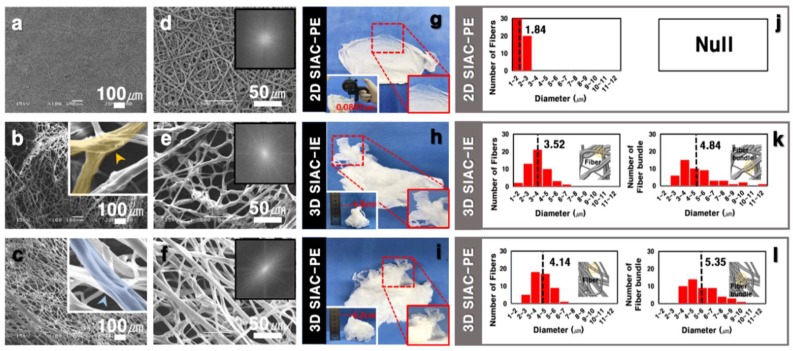
SEM (**a**–**f**) and photographic (**g**–**i**) images of three samples. FFT output images of the corresponding SEM images (inset of **d**–**f**). The thickness of the scaffolds in digital photographs (inset of **g**–**i**). (**j**–**l**) Diameters graphs of fiber and bundle type of each sample.

**Figure 3:**
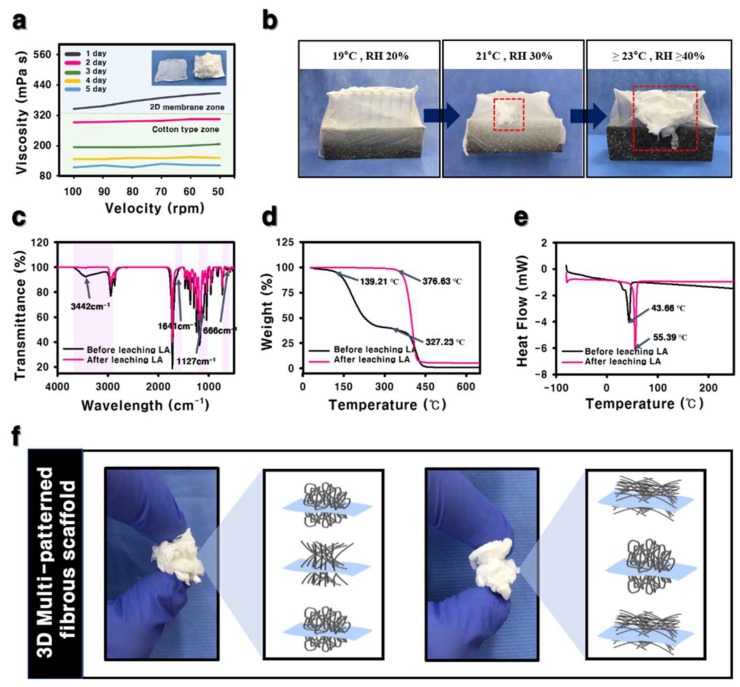
(**a**) Viscosity of PCL/LA solution by increasing mixing time. (**b**) Photographs of fibers dependent on RH and temperature. (**c**) FT-IR, (**d**) TGA, and (**e**) DSC analysis of the fibers (**f**) Photographs of the 3D cotton-type fibrous scaffolds with customized patterns.

The morphology of the fibers fabricated by electrospinning with SIAC-IE and SIAC-PE was investigated, and completely different fiber morphology was confirmed. The as-spun 3D fibers deposited on the SIAC-IE (3D SIAC-IE) was randomly oriented (Figure 2b,e), however, the 3D fibers deposited on the SIAC-PE (3D SIAC-PE) was aligned (Figure 2c,f). When only pristine PCL was used when the electrospinning conditions were not suitable, only 2D mats were fabricated as shown in Figure 2a,d,g. The results demonstrate that the occurrence of different constructs could be because of the difference in surface charge repulsive force between fibers. When residual LA was washed from the PCL/LA fiber for 24 h, the fibers were stable while being immersed in DW. The morphology and alignment of the fibers did not change after LA leaching. Fiber orientation of the samples was evaluated by Fast Fourier Transform (FFT). The aligned fibers and aligned fiber bundles made through the SIAC-PE setting were generally thicker than the other samples (Figure 2l). It also was found that the morphology of the fibers induced by these different electric fields is significantly influenced by the temperature and relative humidity (RH). When the RH and temperature were increased to a critical point (≥40%, ≥23 °C), the electrostatic repulsive force raises and generates the 3D structured self-assembled fibers. However, when the RH is 20% and the temperature is 19 °C or less, the 3D structure is not visible at all, but when they increases to the critical point (≥30%, ≥21 °C), the electrostatic repulsion between the fibers causes the fiber of the 3D structure to start to be generated as shown in Figure 3b. The maximum viscosity to produce a 3D fibrous construct was lower than that to produce a 2D fibrous membrane (Figure 3a). The fiber morphology was found to depend on the viscosity of the solution which varies with the stirring time of LA and PCL solution. For the characterization of the influence of LA in 3D matrixes of before and after the washing process, FTIR, TGA, and DSC analysis were performed as shown in Figure 3c–e. Compared to the fiber of before leaching LA, the characteristic peaks at 3442 and 1127 cm^−1^ corresponding to O-H and C-O stretch of LA, respectively, were not observed in the spectra of the fiber after leaching LA [6,10]. The TGA curve of the fiber of before leaching LA has two stages (first stage: 139–327 °C, second stage: 327–446 °C) of weight loss was observed. However, the fiber of after leaching LA showed a stage of weight loss between 376 and 427 °C (Figure 3d). After leaching LA, the melting temperature was improved from 43.66 °C to 55.39 °C in DSC curve (Figure 3e). These results indicate that LA was clearly washed and only the pure PCL was left.

In contrast to nonspecific deposition by the typical electrospun 3D matrixes, the developed electrospinning method was able to fabricate the 3D fibrous scaffolds with customized patterns. Our work presentes a novel method for the patterning of 3D cotton-type fibrous constructs for the engineering of various tissues, particularly those showing regional differences (Figure 3f). The appropriate pore size and porosity can be crucial for providing adequate space for cell proliferation and allowing for exchange of oxygen and nutrients between the surrounding tissue and matrixes. However, a main problem in conventional electrospinning is the tendency of electrospun fibers to packed densely, showing small pore and poor porosity. The developed 3D electrospun scaffolds presented low packing density and better porosity which can result in improved cell proliferation and infiltration rate compared to 2D tightly packed matrixes. Table 1 presents the results of porosity for the 3D SIAC-IE and 3D SIAC-PE which have different porosity due to the fiber orientation. The effect of the repulsive forces between the fibers containing LA in the electric field forming the aligned fibers was reduced, indicating that the porosity in the 3D SIAC-PE were reduced. However, when compared to 2D electrospun membranes reported in other studies, the porosity of the 3D SIAC-IE and the 3D SIAC-PE increased effectively. Further optimization may enable the preparation of a scaffold suitable for therapeutic applications of 3D target tissues.

**Figure 4:**
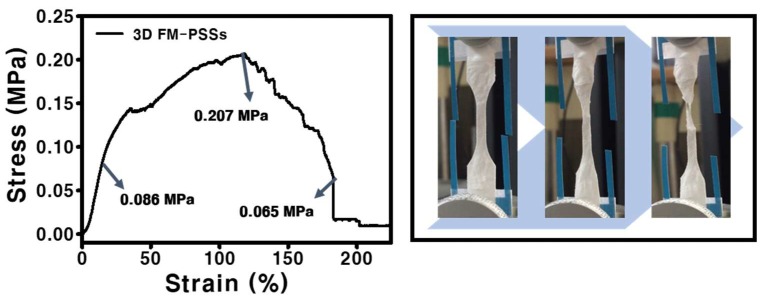
Strain-stress curve and the digital image of 3D FM-PSSs.

The result of the stress-strain curve of the FM-PSS is presented in Figure 4. The FM-PSS exhibited the tensile strength of 0.207 MPa, the yield strength of 0.086 MPa, and the breaking strength of 0.065 MPa. Elongation at break of the scaffold was increased. In addition, the fluctuation in the graph was confirmed due to the existence of interaction between loosely packed fibers.

**Figure 5:**
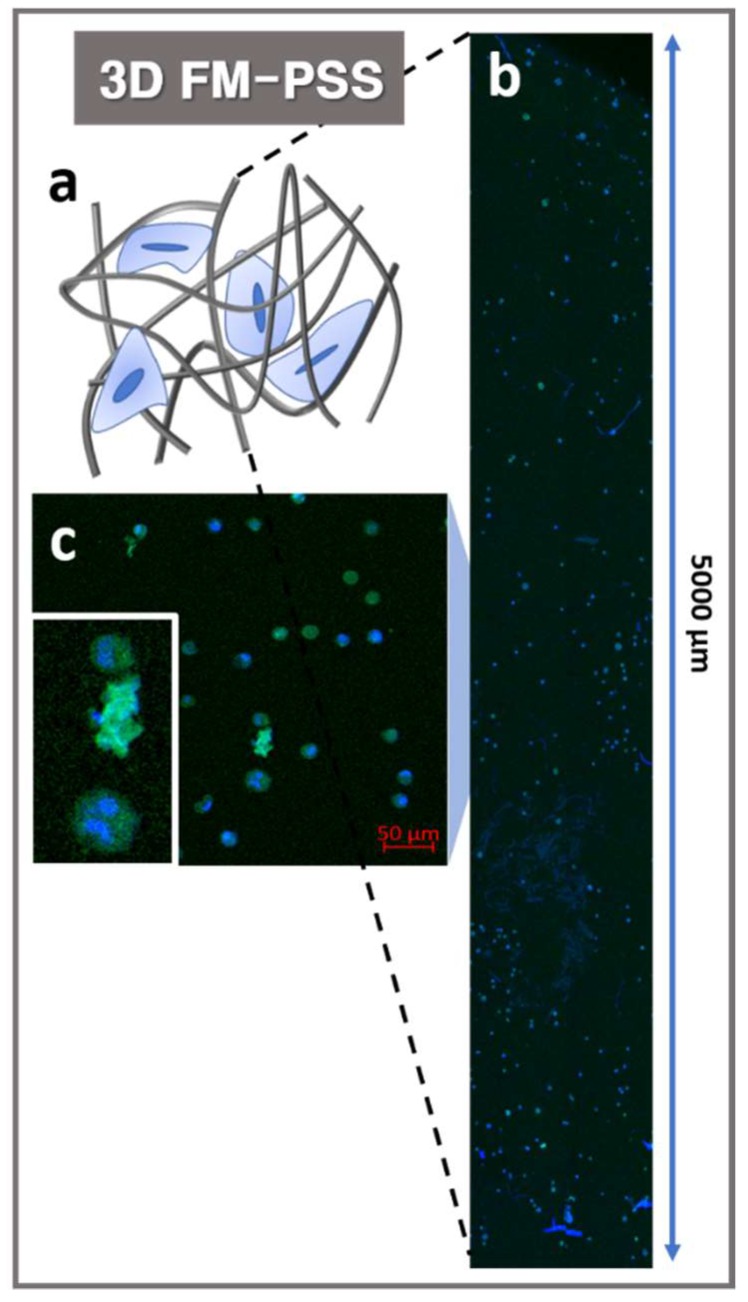
(**a**) Schematic illustration of the cells cultured in 3D FM-PSS. Z-stack image (**b**) and confocal microscopy image (**c**) of the cells on the 3D FM-PSS (inset: the enlarged image).

The electrospun mesh fabricated by conventional electrospinning method would behave as a 2D structured membrane where cells could only migrate on the surface, instead of allowing a 3D construct where cells could infiltrate and proliferate. Cell infiltration into 3D FM-PSS was evaluated using a confocal image processing z-stack program as shown in Figure 5. Each individual image was collected to spectral color-coding step via a z-stack program to add the images with 4′,6-diamidino-2-phenylindole (DAPI) and actin green stained MC3T3-E1 cells. The results of z-stack image confirmed that the MC3T3-E1 cells were well infiltrated and distributed in the 3D FM-PSS as shown in Figure 5.

## 4. Conclusions

In the study, we successfully fabricated the 3D cotton-type fibrous scaffold with customized patterns by a novel electrospinning method. The effect of the electrode shape on the morphology of the deposited fibers’ construct was investigated. In addition, we reported the effect that RH and temperature have on the deposition of the constructs of the developed scaffold due to the change of the number of dissociated ions which can alter the fiber repulsion force. The desired fiber morphology controlled by the shape of the electrodes enables the 3D FM-PSS to be a promising candidate in tissue engineering applications.

## Figures and Tables

**Table 1:** Comparison of porosity measurement results of 3D SIAC-IE and 3D SIAC-PE.

	Total Area (μm^2^)	Pore Area (μm^2^)	Porosity (%)
3D SIAC-IE	48,199	18,242	38
3D SIAC-PE	48,199	8248	17

## Supplementary Materials

The following are available online at https://www.mdpi.com/2073-4360/11/9/1444/s1.
