# Supplementary material for: Harnessing the Topography of 3D Spongy-Like Electrospun Bundled Fibrous Scaffold via a Sharply Inclined Array Collector
Source: Polymers (Basel). 2019 Sep 3;11(9):1444. doi: 10.3390/polym11091444 (PMC6780350; doi:10.3390/polym11091444)
Supplement: Supplementary file 1 [file polymers-11-01444-s001.pdf]

Supporting Information

Harnessing the topography of 3D spongy-like electrospun bundled fibrous scaffold via a sharply inclined array collector

Sun hee Cho[1†], Jeong In Kim[1†], In Gi Kim[1, 2], Chan Hee Park[1, 3]*, Cheol Sang Kim[1, 3]*

[1]*Department of Bionanosystem Engineering, Graduate School, Chonbuk National University, Jeonju 561-756, Republic of Korea*

[2]*Institute of Medical Device Research and Development, TDM Co.,Ltd., 69, Cheomdan venture so-ro 37beon-gil, Buk-gu, Gwangju 61003, Republic of Korea*

[3]*Division of Mechanical Design Engineering, College of Engineering, Chonbuk National University*

*Corresponding authors: Tel.: +82-63-270-4284; fax: +82-63-270-2460

E-mail: chskim@jbnu.ac.kr (C. S. Kim), biochan@jbnu.ac.kr (C.H. Park)

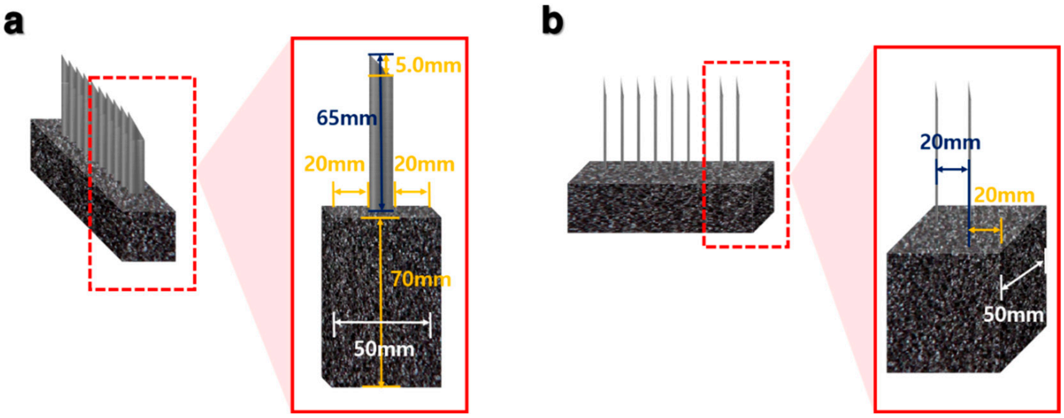

Fig. S1. Illustration of SIAC consisting of nine edged bars.

The electrospinning set up contains a device of the sharply inclined array collector (SIAC) which consists of the nine same edged bars (thickness 0.37mm, length 65 mm) and pedestal. The detailed morphological information of the edged bars and pedestal is shown in Fig. S1.

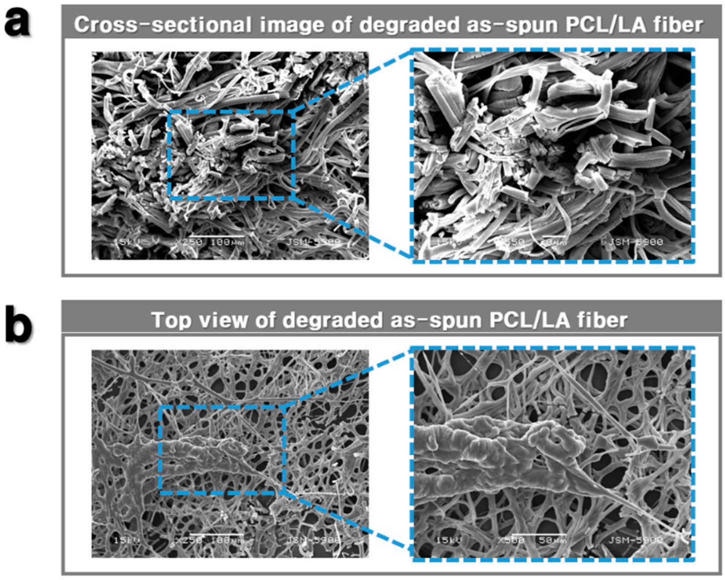

Fig. S2. SEM images of degradation of as-spun PCL/LA fiber.

The morphological variation of the as-spun PCL/LA fiber with residual LA was shown in Fig.S2. The LA leaching process is required to preserve the physical properties of the as-spun PCL/LA fiber.

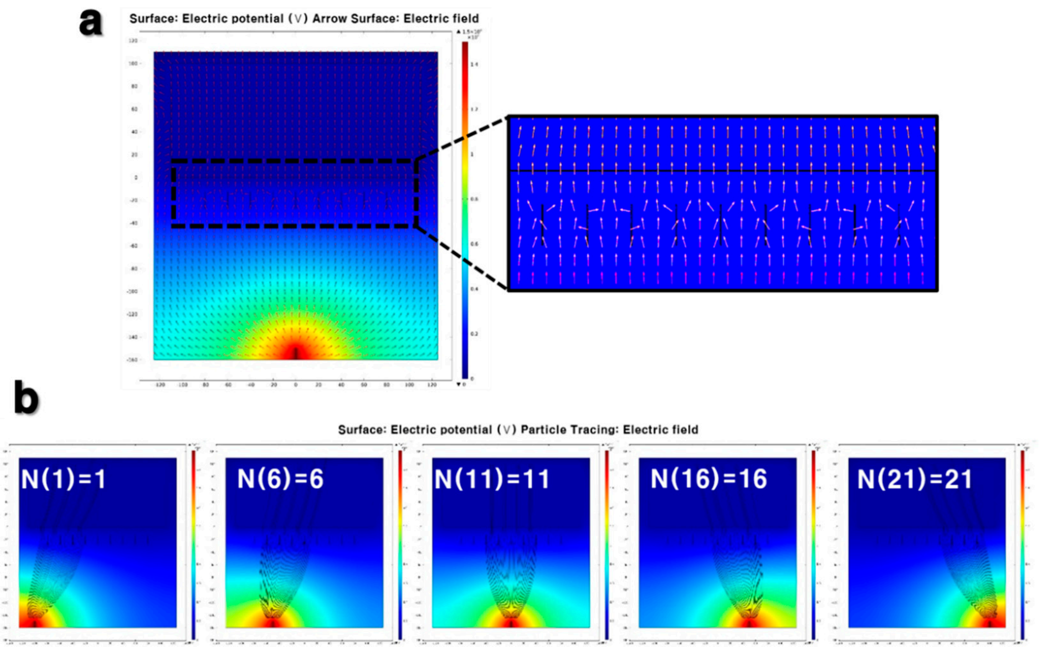

Fig. S3. (a) Distribution of the electric field between needle tip and SIAC. (b) Electric field lines in accordance with needle movement simulated via COMSOL® Multiphysics.

In the electric field distribution analysis results of the setting SIAC with point electrode (SIAC-PE), the electric field was concentrated on the portion where the 3D fiber was generated (middle portion of SIAC), regardless of the movement of the needle tip during electrospinning.

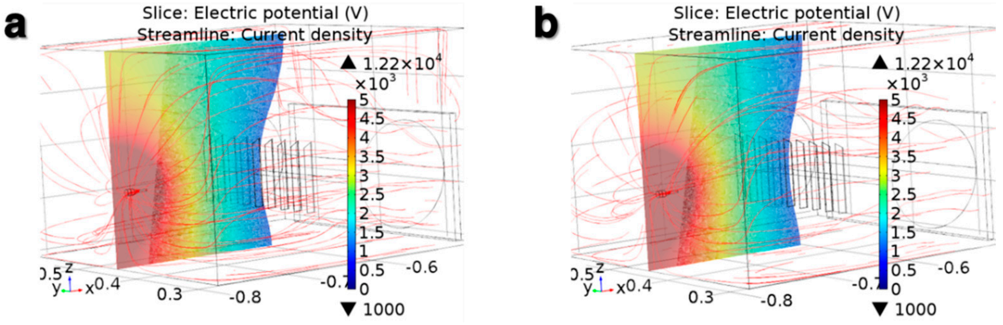

Fig. S4. Electrical streamline simulation result of (a) SIAC-IE and (b) SIAC-PE

In this study, by controlling the position of the edged bars, so-called “point electrode” and “inclined electrode” configurations are designed. According to the results of electrical streamline simulation, it was confirmed that, in the case of the former, the electrical streamlines are drawn toward the point electrodes, whereas, in the latter configuration, the electrical stream lines spread out.
